# Computational analysis of mechanical stress in colonic diverticulosis

**DOI:** 10.1038/s41598-020-63049-w

**Published:** 2020-04-07

**Authors:** Bhavesh Patel, Xiaomei Guo, Jillian Noblet, Sean Chambers, Hans Gregersen, Ghassan S. Kassab

**Affiliations:** 1 California Medical Innovations Institute, San Diego, CA USA; 2 Cook Medical, Inc., Bloomington, IN USA

**Keywords:** Computational models, Computational methods

## Abstract

Diverticulosis results from the development of pouch-like structures, called diverticula, over the colon. The etiology of the disease is poorly understood resulting in a lack of effective treatment approaches. It is well known that mechanical stress plays a major role in tissue remodeling, yet its role in diverticulosis has not been studied. Here, we used computational mechanics to investigate changes in stress distribution engendered over the colon tissue by the presence of a pouch-like structure. The objectives of the study were twofold: (1) observe how stress distribution changes around a single pouch and (2) evaluate how stress elevation correlates with the size of the pouch. Results showed that high stresses are concentrated around the neck of a pouch, and their values and propagation increase with the size of the pouch neck rather than the pouch surface area. These findings suggest that stress distribution may change in diverticulosis and a vicious cycle may occur where pouch size increases due to stress elevation, which in turn elevates stress further and so on. Significant luminal pressure reduction would be necessary to maintain stress at normal level according to our results and therapeutic approaches aimed directly at reducing stress should rather be sought after.

## Introduction

Diverticulosis is a is characterized by the development of pouch-like structures over the colon called diverticula. True prevalence of the disease is difficult to establish since it is mostly asymptomatic and most common symptoms, such as abdominal pain, fever, and diarrhea, are not unique to this condition and often overlooked. Several studies have, however, reported an increase of prevalence with age, with over a third of the population above the age of 60 believed to be afflicted in developed countries^1,2^. When these pouches become inflamed, the disease is commonly termed as diverticulitis^3^, which is the leading cause of lower gastrointestinal bleeding in the United States^4^. Studies have reported between 4 to 20% rate of diverticulitis among asymptomatic diverticulosis patients^5,6^. The incidence of diverticulitis in the United States has reportedly increased by 50% between 1990–1999 and 2000–2007^7^.

Diverticula are structurally characterized by the protrusion of the mucosa and submucosa layers through the muscle layer. Multiple risk factors are implicated for the disease^8,9^ but the actual etiology is not fully understood. It is commonly thought that high colonic pressure resulting from a low-fiber diet leads to the development of diverticula at locations where the colon tissue is likely to be the weakest; i.e., where blood vessels perforce the tissue. Yet, there is no validated theory to support these claims. Since the disease is mostly asymptomatic, it is usually detected unexpectedly (e.g., during a routine colonoscopy). A high-fiber diet is typically prescribed at first to reduce intracolonic pressures. For chronic complications, resection of the infected portion of the colon is currently the only long-term solution^10–12^. A better understanding of the progression of the disease may enable more effective and less invasive treatment approaches to address the disease.

It is well known that mechanical stress is a major factor driving tissue remodeling^13,14^. To the best of our knowledge, the role of stress in diverticulosis has not been investigated. The objective of this manuscript was to use computational structural mechanics to investigate potential changes in stress distribution that could be introduced in the colon tissue due to the presence of a pouch-like structure similar to a diverticulum. Understanding the role of stress could provide a rationale for new therapeutic approaches. To that end, we developed a computational model for the colon based on the mechanical behavior of swine descending colon. The Finite Element (FE) method was used to simulate inflation of normal colon model and colon models with a pouch of various sizes. Stress distribution was observed across the different models and correlation between pouch size and stress elevation was investigated.

## Results

### Validation of the material model implementation with the normal colon model

**Figure 1:**
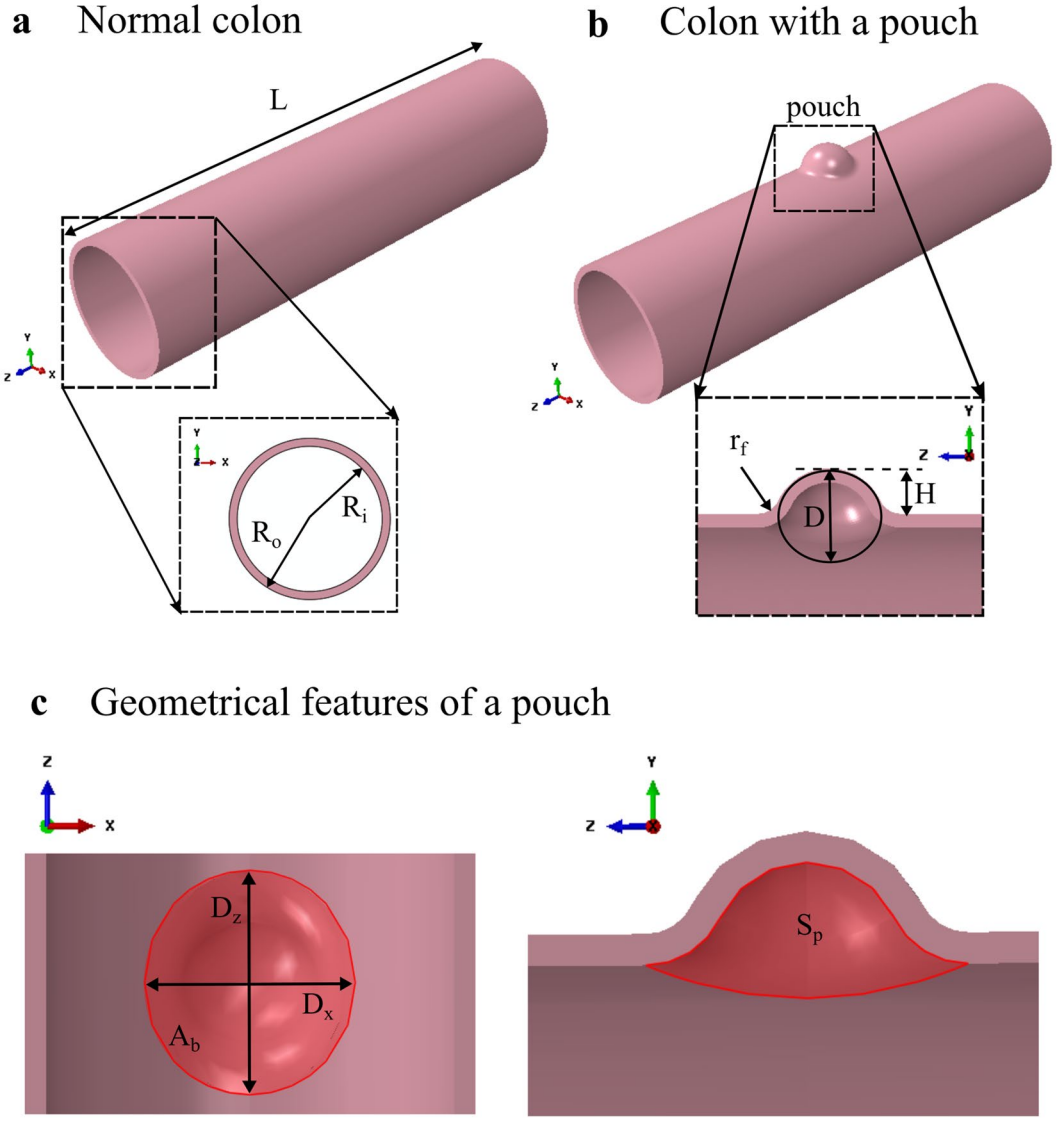
Illustrations of normal colon model (**a**) and diseased colon model with a pouch (**b**). Geometrical features of the pouch that are used to investigate correlation with max principal stress are also shown (**c**): the width of the pouch neck ${D}_{x}$ along the $x$ axis, the width of the pouch neck ${D}_{z}$ along the $z$ axis, the area at the base of the pouch neck ${A}_{b}$, and the surface of the pouch on the lumen side ${S}_{p}$.

**Figure 2:**
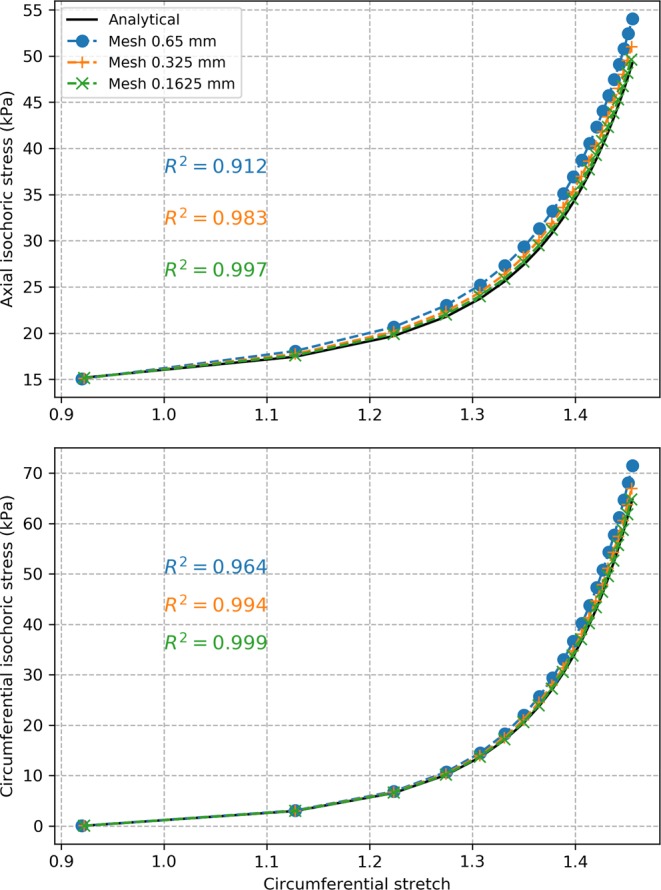
Evolution of isochoric axial and circumferential stresses on the outer surface of the normal colon for the set of material parameters #1 with different mesh sizes.

Geometrical and material properties of swine descending colon tissues were used for the models. In our previous study of swine tissue behavior, we established the first anisotropic material constitutive model for the descending swine colon based on simultaneous inflation and extension tests^15^. This material constitutive model was used in the simulations. The normal colon was modeled as a cylindrical segment (Fig. 1a). The model was simulated with five sets of material parameters, listed in Table 1, resulting in five simulated cases for the normal model. Since the material constitutive model was implemented for the first time in this work in the FE software used, the computational results were validated. First, we looked at convergence of the computational results for successive mesh refinement. The prescribed mesh size was successively divided by a factor of 2, starting from 1.3 mm (value suggested by the FE software), until variation of the maximum value of the max principal stresses from one mesh to the next was less than 10%. This occurred for all simulated normal cases at a prescribed mesh size of 0.325 mm, as changes in the maximum value of the max principal stresses were less than 7% for a further reduction to a mesh size of 0.1625 mm. Then, we checked accuracy of the predicted stress values against analytical results expected for the inflation and extension of a cylindrical tube. At the mesh size of 0.325 mm, average values over all five simulated cases of $0.964\pm 0.033$ (mean $\pm$ s.d.) and $0.982\pm 0.015$ were obtained for the coefficients of determination ${r}^{2}$ between model and analytical results for axial and circumferential isochoric stresses, respectively, suggesting accurate model estimations with this mesh size. Plots of isochoric axial and circumferential stresses on the outer surface of the colon obtained for the normal colon model with the set of material parameters #1 are presented in Fig. 2 for the various FE mesh sizes. Expected analytical values are also presented. These results showed that the material constitutive model was implemented properly in the FE software.

**Table 1:** Material parameters used in the computational model.

Parameters	${C}_{10}$ (Pa)	${k}_{1}^{l}$ (Pa)	${k}_{2}^{l}$	${k}_{1}^{s}$ (Pa)	${k}_{2}^{s}$	$\gamma$ (°)
#1	411	14168	0.63	3016	4.22	44
#2	750	28600	4.55	609	16.94	55
#3	187	33290	2.22	14414	9.33	56
#4	118	7201	1.21	2379	19.97	50
#5	159	1150	1.15	4393	9.82	49

They were established in a previous study for five swine descending colon samples^15^.

### Diseased colon models

#### Stress distribution

**Figure 3:**
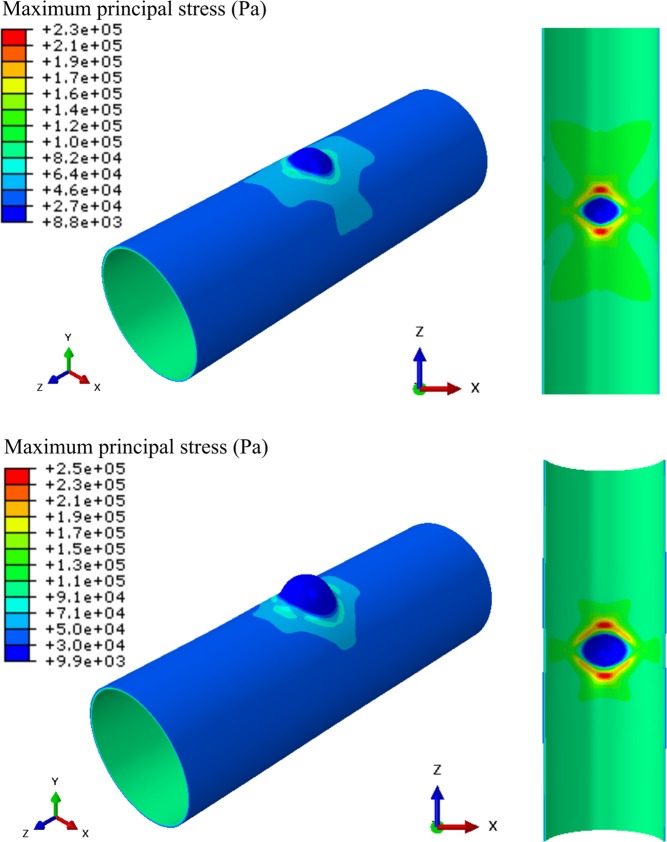
Contour plot of max principal stress with the set of material parameters #1 at a luminal pressure of 3 kPa for a pouch with diameter $D=10$ mm and height $H=4$ mm (top) and a pouch with diameter $D=12$ mm and height $H=6$ mm (bottom). The 3D models are shown (left) along with a view cut across the $XZ$ plane to visualize the lumen side of the pouch (right). High stress values (red regions) are seen around the neck of the pouch on the lumen side.

**Figure 4:**
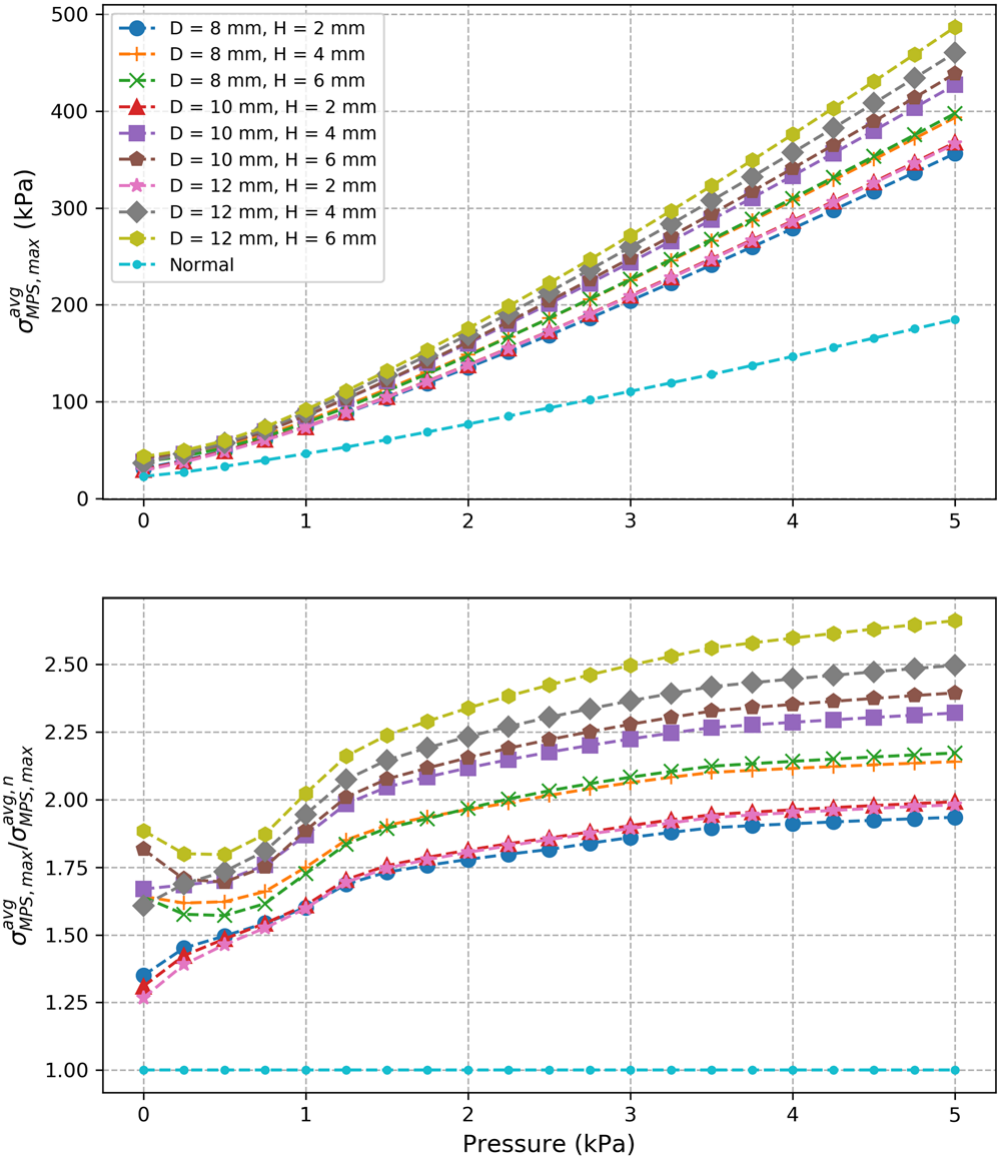
Evolution for each model of the average (over all 5 sets of material parameters) of the maximum value of the max principal stresses ${\sigma }_{MPS,\,max}^{avg}$ (top) and corresponding relative values with respect to normal colon model (bottom).

For the diseased colon models, a diverticulum-like structure was modeled by incorporating a curved protrusion at the center of the normal colon (Fig. 1b). To cover a variety of pouch sizes, three values were considered in the simulations for the initial pouch height $H$ and initial pouch diameter $D$: $H=2$ mm, 4 mm, and 6 mm, and $D$ = 8 mm, 10 mm and 12 mm. Each of the 9 diseased models (resulting from different combinations of $D$ and $H$) was simulated with the five sets of material parameters from Table 1 resulting in five simulated cases for each diseased model. Contour plots of the max principal stress distribution over the colon at a luminal pressure of 3 kPa with the set of material parameters #1 are shown in Fig. 3 for the case of a pouch with diameter $D=10$ mm and height $H=4$ mm and the case of a pouch with diameter $D=12$ mm and height $H=6$ mm. High stress values can be seen around the neck of the pouch on the lumen side. This was consistent throughout all simulated cases and for all pressure values. The evolution of ${\sigma }_{MPS,\,max}^{avg}$, the average over all five simulated cases of the maximum value of the max principal stresses for each diseased model, is shown in Fig. 4. It was observed to increase with pressure. Normalized values with respect to the corresponding value for the normal model ${\sigma }_{MPS,\,max}^{avg,\,n}$ at the same pressure are also shown. The normalized values were always greater than 1 showing that maximum stress values are higher than normal in diseased models.

#### Correlation with pouch geometry

**Figure 5:**
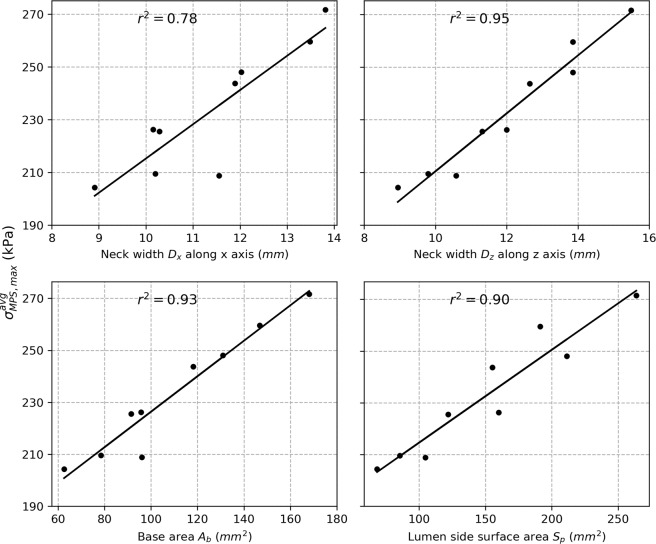
Analysis of the correlation of ${\sigma }_{MPS,\,max}^{avg}$ at 3 kPa against the width of the pouch neck ${D}_{x}$ along the $x$ axis (top left), the width of the pouch ${D}_{z}$ neck along the $z$ axis (top right), the area at the base of the pouch neck ${A}_{b}$ (bottom left), and the surface area of the pouch on the lumen side ${S}_{p}$ (bottom right).

The values ${\sigma }_{MPS,\,max}^{avg}$ for each model were correlated to different parameters of the pouch geometry (Fig. 1c) to identify relation between pouch geometry and high stress. The average ${r}^{2}$ over all pressures considered (between 1 and 5 kPa) and corresponding two-tailed p-values are summarized in Table 2. Analysis of the correlation of ${\sigma }_{MPS,\,max}^{avg}$ at 3 kPa is shown for illustration in Fig. 5. Overall, ${\sigma }_{MPS,\,max}^{avg}$ correlated best with the width of the pouch neck along the $z$ axis ${D}_{z}$ (average ${r}^{2}=0.945$) and the correlation was significant (two-tailed $p < 0.0001$ for all pressures).

**Table 2:** Average r^2^ over all pressures considered and corresponding two-tailed p-values for the correlation of the average maximum value of the max principal stresses with the width of the pouch neck ${D}_{x}$ along the $x$ axis, the width of the pouch neck ${D}_{z}$ along the $z$ axis, the area at the base of the pouch neck ${A}_{b}$, and the surface area of the pouch on the lumen side ${S}_{p}$.

	${D}_{x}$	${D}_{z}$	${A}_{b}$	${S}_{p}$
Average r^2^ over all pressures	0.779	0.945	0.932	0.894
Maximum two-tailed p-value over all pressures	0.00309	0.00007	0.00013	0.00047

#### Pressure factor

We looked at the amount by which a given pressure ${P}_{n}$ must be reduced so that ${\sigma }_{MPS,max}$ at the reduced pressure is similar to ${\sigma }_{MPS,\,max}^{n}$ at ${P}_{n}$. We designated the associated pressure drop by pressure factor. The average pressure factor for all simulated cases was seen to vary between 40% and 56% for ${P}_{n}$ in the range 2 to 5 kPa.

#### Zone of influence around a pouch

**Figure 6:**
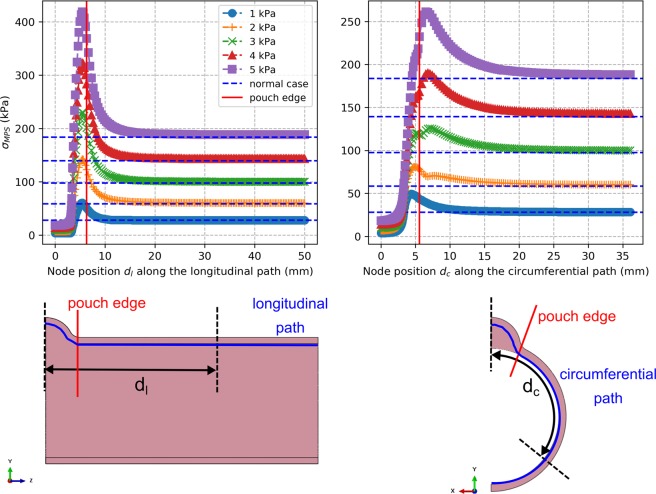
Evolution of the max principal stress ${\sigma }_{MPS}$ on the lumen side surface along the longitudinal and the circumferential paths passing through the center of the pouch for the model with pouch diameter $D=10$ mm and height $H=4$ mm and with the set of material parameters #1 (top row). The paths and the measurements of the node position ${d}_{l}$ along the longitudinal path and node position ${d}_{c}$ along the circumferential path are illustrated on drawings of the model (bottom row). Only a quarter of the model was used for analysis due to symmetry.

**Figure 7:**
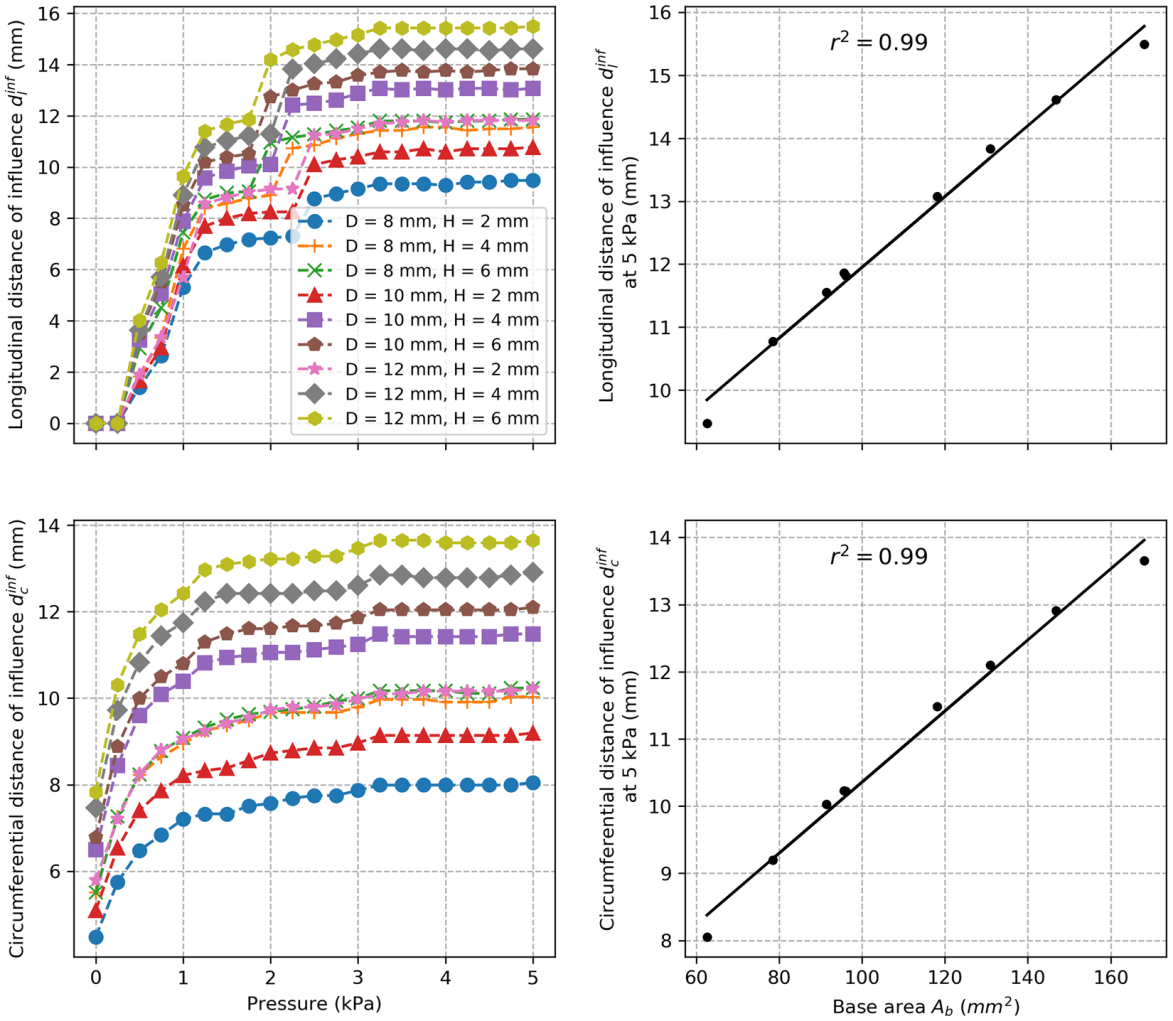
Evolution of the average longitudinal and circumferential distances of influence for each model (left column) and the correlation of their plateau values (i.e., value at 5 kPa) with the area at the base of the pouch neck ${A}_{b}$ (right column).

The evolution of the max principal stress on the lumen side surface along the longitudinal and circumferential paths passing through the center of the pouch is shown in Fig. 6 for the case of a pouch with a diameter $D=10$ mm and height $H=4$ mm and with the set of material parameters #1. For all cases and at all the different pressures, we observed that stresses are elevated near the edge of the pouch, then reduces towards normal levels as we move away from the edge. To investigate how far from the pouch stress is actually elevated, we established a zone of influence: we calculated the distances ${d}_{l}^{inf}$ and ${d}_{c}^{inf}$ from the center of the pouch after which max principal stress in the diseased models drops within 10% of normal level along the longitudinal and circumferential paths, respectively. Average values over all cases for each simulated diseased model are presented as a function of luminal pressure in Fig. 7. We observed that the distances of influence increase with pressure before reaching a plateau level. The average plateau values of ${d}_{l}^{inf}$ and ${d}_{c}^{inf}$ for each model range between 8 to 16 mm for the pouch sizes considered in this work. After correlating these average values with the parameters of the pouch geometry, we found that both correlated most with the area at the base of the neck ${A}_{b}$ (${r}^{2}=0.99$) and the correlation was significant (two-tailed $p < 0.0001$).

## Discussion

Computational models have been established previously for the normal colon tissue. Most are, however, based on isotropic material models^16–18^ despite the anisotropic nature of the colon tissue. One study implemented an anisotropic computational model based on uniaxial testing^19^. To our knowledge, this is the first anisotropic computational model of the colon based on simultaneous inflation and extension data. For five sets of material parameters derived previously with swine descending colon tissues, the model was validated against analytical results expected for a cylindrical tube. Convergence was verified through mesh refinement and correlation with analytical values of ${R}^{2}=0.964$ and $0.982$ were obtained on average for axial and circumferential isochoric stresses, respectively, at the mesh size retained. Due to the lack of available data in the literature, an idealized geometry was assumed for the pouch intended to represent a diverticula-like protrusion. Despite the idealized geometry, this is the first attempt to study variation of stress distribution over the colon due to the presence of a pouch-like structure. Analysis with various pouch sizes revealed that stress values higher than normal are seen in the diseased models with a pouch. Especially, highest stress values are seen to be concentrated around the neck of the pouch on the lumen side. Stress elevation in the diseased models was seen to increase with pressure and the maximum value of the max principal stresses could reach up to 2.8 times the maximum values seen the normal colon model. This shows that a significant elevation of stress could take place in a colon with diverticulosis compared to normal colon and implicates elevated stresses in this condition. For any given luminal pressure value, the maximum value of the max principal stresses was found to correlate well with parameters of the pouch geometry and corralled most with the width of the pouch neck. The analysis shows that there is correlation between stress elevation and size of the pouch. Since it is known that pouch size increases overtime in diverticulosis and that mechanical stress is a major factor driving biological tissue remodeling, our findings thus suggest a novel hypothesis for the mechanism of diverticula progression where the overall pouch size may increase in response to elevated stress values around the pouch. This in turn may further increase these stress values leading to a vicious cycle where the pouch size is further increased. Moreover, we found that the zone of influence of a pouch, i.e. distance from the center of the pouch after which stress drops within 10% of the normal model, also increases with pressure. It reaches a plateau value after a certain pressure elevation. This value ranged between 8 to 16 mm for the simulated cases. This shows that, in the cases of multiple pouches, pouches located within twice this distance may interact mechanically or otherwise may act as a single pouch. The plateau value of the distance of influence correlated best with the area of the pouch neck. Overall, these findings also suggest that the size of the pouch neck is more important than the surface area of the pouch when identifying pouches under high stress and with a greater zone of influence. Moreover, our simulations show that a significant luminal pressure drop (between 40% and 56% for the simulated cases) would be necessary to restore stress back to a normal level. This would explain the low effectiveness of a high-fiber diet as a stand-alone treatment solution once pouches are developed^20–22^ since it may not be enough to achieve the required pressure drop to reduce stress in the tissue and prevent pouch progression.

There are some limitations to the model established in the present study. We assumed the pouch material to be similar to the normal colon although a diverticulum is expected to be more compliant than normal tissue – it is constituted by the mucosa and submucosa layers, that are known to be more compliant than the muscle layer. Indeed, the mucosa has been reported to be extremely expansible^23^ and it has been shown that the submucosa could withstand deformations 4 to 5 times greater than the muscle layer^24^. This is, however, a conservative approach since stress elevations are expected to be even greater with a more compliant pouch material due to compliance mismatch between normal and pouch materials. Use of planar biaxial tests^25–29^ on portions of normal colon tissue and pouch tissue excised from the same colon sample should be considered in future studies to account for variation in material properties. The present model is static that considers snapshots of disease progression through different pouch sizes in order to investigate the correlation between stress level and pouch size. While beyond the scope of the current study, a dynamic model accounting for pouch growth and remodeling should be considered in future studies to understand the relation between stress level and temporal evolution of the pouch size. This effort will require morphological and mechanical data from diseased tissue with diverticula at multiple stages of the development of the disease. While some data is available^30–32^, it is limited and not fully suitable to develop a proper growth and remodeling model of pouch progression without ad hoc assumptions. Even the simplest dynamic model would require knowing, at multiple time points, pouch size, boundary conditions (inner and outer wall pressures), and change in mechanical properties of the isolated pouch tissue. To the best of our knowledge, no such data are currently available. While mathematical and computational tools could certainly be borrowed from other applications to account for all the above-mentioned aspects, data from normal as well as diseased colon tissues is required to inform and validate more complex models. We recently established the first large animal model of diverticulosis^33^ that can be used to collect such data. Finally, it is important to mention that the colon is a collapsible tube with curves. A simplified geometry was assumed here as a starting point to model a segment of the colon tissue. With tortuosity and curvature accounted, the stress values would most certainly change but we expect that the overall analysis will remain unchanged, i.e., higher stress would be observed at the neck of the pouch with a relative increase with pressure and pouch size. The goal here was to focus on single pouch rigorously rather than multiple pouches superficially. Modeling of multiple pouches is the next logical step.

This study provides the first analysis of changes in mechanical stress that may occur in the colon tissue due to the development of a diverticulum. The simulation model lays the foundation for computer-aided analysis in colonic diverticulosis which is currently lacking. The findings suggest for the potential role of mechanical stress in diverticulosis. Approaches aimed directly at reducing mechanical stresses rather than intra-colonic pressure could be sought for more effective treatment. According to our analysis, pouches with a larger neck should be the primary target of such approaches. The proposed model can serve as a virtual platform to assess such therapies.

## Methods

### Colon model

The Finite Element software Abaqus (version 6.13, SIMULIA, Providence, RI) was used to develop the models and conduct associated simulations. All simulations were performed on a Dell Workstation T7810 with two Intel Xeon processors and 32 GBytes of memory. In our previous study of swine tissue behavior, we established an anisotropic material constitutive model for the descending and spiral regions of the swine colon based on simultaneous inflation and extension tests^15^. This model has been shown in several previous studies to capture adequately the anisotropic behavior of the colon^19,34–36^. The material parameters established in our previous study were retained since, to our knowledge, it is the only study modeling a large animal colon under inflation and extension, the two major deformation modes of the colon. Moreover, it has been established that swine colon is relevant for diverticulosis study^33,37^. The selected material model and parameters are thus adequate choices for this study. Since the spiral colon anatomy is not relevant to humans, results from the descending colon samples were used here for characterizing the colon tissue material. We have reported in our previous work that there is negligible variation in diameter locally along the length of a swine descending colon segment thus the normal colon was modeled as a cylindrical segment (Fig. 1a). The inner and outer radius were set to ${R}_{i}=11.5$ mm and ${R}_{o}=12.7$ mm, respectively, based on average values measured in our previous work. The axial length was set to $L=10$ cm such that it is several folds greater than the radius. Specifically, the colon tissue was assumed to be a homogeneous, anisotropic and incompressible hyperelastic material whose mechanical behavior is described by the following anisotropic strain energy function $\bar{W}:$^15,35^

1 $$\bar{W}={C}_{10}({I}_{1}-3)+\frac{{k}_{1}^{l}}{{k}_{2}^{l}}[{e}^{{k}_{2}^{l}{({I}_{4}^{l}-1)}^{2}}-1]+\frac{{k}_{1}^{s}}{{k}_{2}^{s}}[{e}^{{k}_{2}^{s}{({I}_{4}^{s}-1)}^{2}}-1]$$

The first term represents a Neo-Hookean response characterizing the behavior of the non-collagenous constituents of the colon tissue. The quantity ${I}_{1}={\lambda }_{z}^{2}+{\lambda }_{\theta }^{2}+\frac{1}{{\lambda }_{z}^{2}{\lambda }_{\theta }^{2}}$ represents the first invariant of the deformation tensor with ${\lambda }_{z}$ and ${\lambda }_{\theta }$ referring to the axial and circumferential stretches, respectively. The subsequent terms characterize the contribution of the collagen fibers, with ${k}_{1}$ and ${k}_{2}$ being a measure of fiber stiffness. Specifically, the second term (with superscript $l$) characterizes the behavior of the collagen fibers aligned along the longitudinal (axial) direction, while the third term (with superscript $s$) characterizes the response of the collagen fibers dispersed in two preferential symmetric directions with respect to the circumferential direction. The quantity ${I}_{4}$ is referred to as pseudo-invariant and characterizes the mechanical response of the fibers along the preferential directions. For the second term, ${I}_{4}^{l}={\lambda }_{z}^{2}$ since it accounts for the fibers along the longitudinal direction. For the third term, ${I}_{4}^{s}$ is expressed as:

2 $${I}_{4}^{s}={\lambda }_{\theta }^{2}{\cos }^{2}{\gamma }^{s}+{\lambda }_{z}^{2}{\sin }^{2}{\gamma }^{s}$$

Here, $\pm {\gamma }^{s}$ indicates the angle of the two symmetric fiber orientations angle with respect to the circumferential direction. Five sets of material parameters, previously derived for five swine descending colon samples, were used in this work for the material parameters ${C}_{10},\,{k}_{1}^{l},\,{k}_{2}^{l},\,{k}_{1}^{s},\,{k}_{2}^{s},$ and ${\gamma }^{s}$. This strain energy function is not directly available in Abaqus. It was thus integrated for the first time by implementing a Fortran based user subroutine called UANISOHYPER_INV, which allows defining anisotropic hyperelastic material behavior using the invariant formulation. This subroutine required defining the first and second derivatives of the strain-energy function (Eq. 1) with respect to the scalar invariants ${I}_{1}$ and ${I}_{4}$. To verify that the subroutine was implemented properly and validate the computational model of the colon tissue, we compared model-estimated stress values of isochoric circumferential stress ${\bar{\sigma }}_{\theta }^{m}$ and isochoric axial stress ${\bar{\sigma }}_{z}^{m}$ on the outer colon surface against values obtained analytically based on the strain energy function and calculated as follows:

3 $${\bar{\sigma }}_{\theta }^{a}={\lambda }_{\theta }\frac{\partial \bar{W}}{\partial {\lambda }_{\theta }}$$

4 $${\bar{\sigma }}_{z}^{a}={\lambda }_{z}\frac{\partial \bar{W}}{\partial {\lambda }_{z}}$$

The coefficient of determination ${R}^{2}$ was calculated as follows to quantify the deviation between computational and analytical values:

5 $${R}^{2}(A)=1-\frac{{\sum }_{q=1}^{n}{({A}_{q}^{m}-{A}_{q}^{a})}^{2}}{{\sum }_{q=1}^{n}{({A}_{avg}^{a}-{A}_{q}^{a})}^{2}}$$

where $A={\bar{\sigma }}_{z},\,{\bar{\sigma }}_{\theta }$, and the subscript “$avg$” indicates the average of the analytical values over all $n$ data points. A value of ${R}^{2}$ close to 1 indicates that a good correlation is globally obtained.

### Diverticulum model

For the diseased colon models, a diverticulum-like structure was modeled as the intersection of a sphere of diameter $D$ with the cylindrical shape of the normal colon at a height $H$ above the surface. Only one pouch was considered in this study to focus on the effect of a single pouch without additional variabilities that would be incorporated from multiple pouches (number of pouches, positions along the colon, relative position with respect to each other, variability in individual size, etc.). To our knowledge, there is no study systematically reporting the geometry of the diverticula. It is commonly mentioned that they are typically between 5 to 10 mm in diameter without proper indication as to where the diameter is measured^38,39^. To cover a variety of pouch sizes, three values were considered in the simulations for the initial pouch height $H$and initial pouch diameter $D$: $H=2$ mm, 4 mm, and 6 mm, and $D$ = 8 mm, 10 mm and 12 mm. The combinations of these values of D and H led to 9 diseased models with different pouch sizes. A round fillet of radius ${r}_{f}=2$ mm was included at the intersection between the cylinder representing the normal colon and the sphere representing the pouch to remove sharp edges that may not be encountered in the actual tissue and may alter stress values. The thickness of the diverticulum tissue was assumed to be the same as the colon (i.e., ${R}_{o}-{R}_{i}$). There have been attempts to characterize the colon mechanics in diverticulosis^23,30,40,41^. No material properties, however, have been established specifically for the diverticulum tissue (i.e., characterization of the isolated diverticulum pouch). Properties similar to the normal tissue were thus considered for the pouch section.

### Boundary conditions

The boundary conditions were imposed based on our previous study of swine colon^15^. We observed that luminal pressure (pressure on the inner wall of the tissue with outer pressure equal to zero) values above 1.5 kPa led to permanent damage to the tissue under passive conditions. In the computational simulations, this value was pushed further to visualize the trend of the evolution of stress values and luminal pressure up to 5 kPa was imposed on the inner surface of the colon, including pouch. The outer wall pressure was kept equal to 0. Since we are working with a thin-walled tissue (inner diameter to thickness ratio close to 20), the luminal pressure could thus be assimilated as the delta between inner pressure (e.g., pressure imposed inside the conduit of the tissue by fecal matter or intestinal gas) and outer pressure (e.g., abdominal pressure). Typically, a small pressure would be required to open up the colon tissue that would otherwise collapse. That pressure (~0.2 kPa) is, however, very small compared to the range considered here so it is ignored. The pressure was applied in quasi-static increments. Axial stretch of about 10% was typically seen *in vivo* in the colon during our previous study. Thus, a 10% axial stretch was imposed along the $Z$ direction before pressurization. We have found very small opening angle values in the swine colon tissue, and residual circumferential stresses were thus neglected in the model. For both normal and diseased models, we used symmetry with respect to the $YZ$ plane and $XY$ plane and solved only a quarter of the model to achieve higher efficiency, before reconstructing the entire model for visualization purpose.

### Meshing

Appropriate geometry sectioning was done in each case to ensure meshing is possible using linear hexahedral elements. Hybrid element formulation was selected to enforce material incompressibility (i.e., element C3D8H from the Abaqus library). A mesh convergence study was conducted for the normal model by reducing the reference mesh size starting from the default size suggested automatically by Abaqus (1.3 mm) until the variation of the maximum value of the max principal stresses between two successive meshes was less than 10%. The converging mesh size was then also used in the diseased models, with some further refinements around the pouch. A total of 69608 elements and 88350 nodes were used for the normal model at the finest mesh size retained for analysis (quarter of the geometry). Between 69764 elements and 80656 elements (88605 and 102440 nodes respectively) were used for the nine diseased models (quarter of the geometry). A local material orientation was specified using the discrete formulation option available in Abaqus to define properly the fiber orientation in each element as required in the strain energy function.

### Results analysis

A total of 10 models (one normal and nine diseased with different pouch sizes) were considered. Each model was simulated with 5 different sets of material parameters resulting in a total of 50 simulated cases. A measure of stress level is necessary to compare across the models. Since we are not establishing a failure criterion but simply looking at variations under different conditions, any measure such as max principal stress or von Mises stress would be adequate. Results are shown here only in terms of the max principal stress to avoid redundant information since overall observations with von Mises stress were found to be similar. Analysis and results with von Mises stress are included in the data associated with this study if the reader wishes to consult them. For each simulated case, the max principal stress (at the centroid of a mesh element), referred to as ${\sigma }_{MPS}$, was observed in a pressure range of 0 to 5 kPa with an increment of 0.25 kPa. A Python script was developed to automatically search for the maximum value of ${\sigma }_{MPS}$, designated as ${\sigma }_{MPS,max}$ at the various pressures for each simulated case. For each model, the average value obtained for each simulated case (i.e., for all five sets of material parameters), was calculated and retained for subsequent analysis. These values are referred to as ${\sigma }_{MPS,\,max}^{avg}$ and ${\sigma }_{MPS,\,max}^{avg,\,n}$ for diseased and normal models, respectively.

The values ${\sigma }_{MPS,\,max}^{avg}$, were correlated, for pressure between 1 and 5 kPa (in increment of 0.25 kPa), to different parameters of the pouch geometry (Fig. 1c) to identify relation between pouch geometry and high stress: the width of the pouch neck along the $x$ axis ${D}_{x}$, the width of the pouch neck along the $z$ axis ${D}_{z}$, the area at the base of the pouch neck ${A}_{b}$, and the surface of the pouch on the lumen side ${S}_{p}$. The Pearson correlation coefficient ${r}^{2}$ was calculated for each parameter. The two-tailed p-value was calculated as well to evaluate the significance of the correlation.

Moreover, we calculated the pressure ${P}_{d}$ in the diseased models such that for a given pressure ${P}_{n}$ in the normal colon we have:

6 $$\max ({\sigma }_{MPS,max}({P}_{d}))\le \,\max ({\sigma }_{MPS,\,max}^{n}({P}_{n}))$$

The following pressure factor was subsequently computed as:

7 $$f=\left(\frac{{P}_{n}-{P}_{d}}{{P}_{n}}\right)\times 100$$

This factor indicates the % by which the pressure ${P}_{n}$must be reduced in the diseased colon so that ${\sigma }_{MPS,max}$ is similar to ${\sigma }_{MPS,\,max}^{n}$, i.e maximum value of the max principal stress in the diseased model doesn’t exceed the corresponding value in the normal model. The reduced pressure is then designated as ${P}_{d}$. Specifically, for each simulated case, ${P}_{n}$ was reduced by 0.25 kPa until the relation from Eq. 6 was satisfied, providing the corresponding value of ${P}_{d}$ and the pressure factor $f$ was computed. This was done for ${P}_{n}$ in the range 2 to 5 kPa (in increment of 1 kPa). The average value obtained for the models over all simulated cases was calculated and retained for subsequent analysis.

Finally, we established the zone of influence of a pouch, i.e. distance around a pouch where stress is elevated. To achieve that, we established for each diseased case a map of the max principal stress on the lumen side surface of the colon (since stress is highest on this surface) along meshing nodes located on the longitudinal and circumferential paths going through the center of the pouch. The evolution of the max principal stress along these paths was observed as a function of the distances ${d}_{l}$ and ${d}_{c}$ measured with respect to the center of the pouch. Then, we calculated the farthest distances ${d}_{l}^{inf}$ along the longitudinal path and ${d}_{c}^{inf}$ along the circumferential path after which max principal stress drops within 10% of corresponding value in the normal model along the same paths. These distances constituted our measure of the zone of influence. Observing that they reach a plateau value after a certain pressure, their average values over all cases for each model at 5 kPa were correlated to different parameters of the pouch geometry, similarly to ${\sigma }_{MPS,\,max}^{avg}$.

All the calculations were done in Python programming language by developing Jupyter notebooks^42^. Data were stored and manipulated with the Pandas library of Python^43^. Basic mathematical operations were done with the NumPy library^44^. The correlation coefficient ${r}^{2}$ and two-tailed p-value were calculated with the “stats” function of the Scipy library^45^. All the plots were realized with the Matplotlib library^46^.

## Supplementary

Results are presented here in terms of average values over the simulated cases for each model and specific cases are presented for illustration purposed. Individual results for all simulated cases are, however, available in the Excel files included in the datasets of this work. Jupyter notebooks, created to manipulate and visualize results, are also included in the datasets. The Abaqus model (.cae) and input (.inp) files for all models and simulated cases are available as well for the readers to reproduce/modify the simulations and results presented here. The datasets generated and analyzed during the current study are publicly available at the NIH SPARC Portal (10.26275/duz8-mq3n) for normal model analysis and in the associated Zenodo repository (10.5281/zenodo.3611633) for diseased models. A protocol has been created to explain the steps for running the simulations and it is available on protocols.io (10.17504/protocols.io.wzeff3e).
